# Supplementary material for: Chromothripsis during telomere crisis is independent of NHEJ, and consistent with a replicative origin
Source: Genome Res. 2019 May;29(5):737–49. doi: 10.1101/gr.240705.118 (PMC6499312; doi:10.1101/gr.240705.118)
Supplement: Supplemental Material [file supp_gr.240705.118_Supplemental_file_1.zip › contigs/annotated_contigs/DB57/contig.2.DB57_length_740_mean_cov_10.3378378378.docx]

**DB57_length_740_mean_cov_10.3378378378**

```
TGTTTGTTTGAAAAAGACTATGCCTTTCCTTCATATATGTTGCTTAGTTTTGCTGGATACAGAATTGTTCGCTGATAATTGTTTTGTTT
 >chr1:193243830-193244156 + E=4e-177
GAGGAGGCTGAAGATAGGGCCCCAATCCTTTCTAGCTTGTAGGGTTTCTGCTGAGAAATCTGCTGTTAATCTGATAGGTTTTCCTTTAT

AGGTTACCTGGTGCTTGTGTCTCACAGCTCTTAAGATTCTTTCCTTTGTCTTAACTTTGGATAATCTGATGACAGTGGGCCTAGGTGAT

GATCTTTTTGTGATGGATTTCCCAGATGTTCTTTGTGCTTCTTGTACTTGTATTTGCATGTCTAG|TACAT|GAAAATCCATGTATAAA
                                                                       >chr1:193208043-
TGAACCCACTCAATTGGAACTTGTATTGTTCAAGGATCAAATGTATATGGTTATGTCCCTATTTCTCTTCTCCCATAATTTTAAATACC
193208446 + E=1e-230
CTCTCAAGAAGATCTCATAGCTTTCAATTACCATTTATATTCCCTCATTCTAAAAATTATGTCTTTAGCTCAATATTCTGGACCTATAT

ACCTCACTCACTATGAGATAACTTCTCTGTGATATCTCATTGGTACATCAAACTCAACCTTTTCCAAATGGAATTGATGGTTTTACTCT

CTTCCTTATTCCTCAAACTATTTCTTATTATCTGTTAGTCCTTTTCAGTAATTTGCCTAAGCCAGAAACTTGGGAGTATATTATCTTAG

CTCTTGGTTGATCTTTCTGCTTTCACTCTT
```
